# Editorial: The role of transcription factors, stem cell markers and epigenetics contributing to chemoresistance in brain cancers

**DOI:** 10.3389/fonc.2023.1263469

**Published:** 2023-09-01

**Authors:** Javier S. Castresana, Mehdi H. Shahi, Ashok Sharma

**Affiliations:** ^1^ Department of Biochemistry and Genetics, University of Navarra School of Sciences, Pamplona, Spain; ^2^ Interdisciplinary Brain Research Centre, Faculty of Medicine, Aligarh Muslim University, Aligarh, India; ^3^ Department of Biochemistry, All India Institute of Medical Sciences, New Delhi, India

**Keywords:** transcription factor, stem cell marker, epigenetics, chemoresistance, brain tumors

This Research Topic considers possible new advances regarding the contribution of transcription factors, stem cell markers and epigenetic regulation to the phenomenon of resistance to chemotherapy in brain tumors. Glioblastoma and medulloblastoma are the most common malignant brain tumors, typically occurring in adults and children, respectively. Talking today, in general, about glioblastoma or medulloblastoma is almost meaningless, since these tumors present great cellular and molecular heterogeneity that complicates the therapeutic response. For this reason, it is very important to continue investigating their molecular etiology: any advance in the knowledge of the multiple molecular subtypes of brain tumors will undoubtedly lead to the development of new treatments that can be directed specifically against these molecular targets.

Li and Gao demonstrated that a long non coding (lnc) RNA (Linc00883) promotes drug resistance in glioblastoma cells. To do that, they induced ectopic expression of Linc00883 in glioma cells, which resulted in an increase of cell proliferation, a decrease in cell apoptosis and an increase of MRP (multidrug resistance-associated protein) which was then associated to an increase of chemoresistance. Therefore, overexpression of Linc00883 was associated to a bad prognosis for glioma. The authors studied the possible relationship among Linc00883, miR-136 and NEK1 expression.

Linc00883 had previously been shown to increase colorectal cancer cell proliferation, invasion, and migration by regulating the miR-577/FKBP14 axis (1). On the contrary, miR-136 seems to act as a tumor suppressor miRNA. miR-136 inhibited tumorigenesis *in vitro* and *in vivo* by targeting KLF7 in glioma (2); increased sensitivity to temozolomide in glioma cells by targeting AEG-1 (3), promoted apoptosis of glioma cells by targeting AEG-1 and Bcl-2 (4), and reversed cisplatin resistance and enhanced the response to cisplatin treatment by targeting E2F1 in glioma cells (5).

The recent discoveries on the epigenetic regulation of gene expression, linking lncRNA with miRNA, present a new scenario in which miR-136 inhibition is itself inhibited by lncRNAs that act as oncogenic regulatory molecules. There are various examples of this interaction of non-coding RNAs -lnc and miRNA-, in cancers such as endometrial (6), cervical (7), triple-negative breast cancer (8) and bladder cancer (9), among others.

The serine/threonine NEK kinases are encoded by the Never In Mitosis A (NIMA) gene and play a role in the cell cycle, checkpoint regulation, and primary cilia biology. There are eleven members, namely NEK1 to NEK11, that regulate specific events of mitosis, like centrosome separation, spindle assembly and cytokinesis (10–13). Nek1 is overexpressed in gliomas (14). A great effort is underway in producing NEK1 inhibitors that might reduce cancer cell proliferation (11). For example, Nek1 protein inhibitor (iNek1) and TMZ decreased cell viability and tumour size in glioblastoma (15).

The strategy presented by Li and Gao in this Research Topic, evaluates the epigenetic regulation of NEK1 by a tumor suppressor miRNA (miR-136) and an oncogenic lncRNA (Linc00883). The competitive binding of Linc00883 to miR-136 reduces the binding of miR-136 to NEK1, and therefore, NEK1 expression is increased, then inducing an increase of cell proliferation and drug resistance, while cell apoptosis is reduced. Linc00883 can be taken, in this way, as a potential prognostic biomarker and/or therapeutic target in glioma.

Secondly, Geng et al. presented an interesting study in this Research Topic showing the contribution of the miR-137-XIAP axis together with TRAIL, to induce apoptosis in glioblastoma. XIAP is an X-linked inhibitor of caspases and, therefore of apoptosis (16). Recent research aimed at discovering XIAP inhibitors has become important for cancer therapy. When miR-137 is expressed sufficiently, XIAP expression is suppressed, and then, apoptosis of glioblastoma cells is not inhibited. The authors (Geng et al.) demonstrated that miR-137 sensitized glioblastoma cells to TRAIL-mediated apoptosis. Furthermore, the co-treatment of miR-137 and TRAIL potently suppressed glioblastoma tumor growth *in vivo*. This makes miR-137 a good candidate to be highly expressed in glioblastoma cells in order to sensitize them to TRAIL-mediated apoptosis.

Thirdly, in this Research Topic, Gu et al., show that DACH1 (Dachshund Family Transcription Factor 1) expression was significantly downregulated in temozolomide-resistant cells. It is well accepted that DACH1 acts as a tumor suppressor gene in glioma cells (17, 18). The new result by Gu et al. linking DACH1 downregulation to temozolomide resistance is of importance in order to try to maintain DACH1 expression – thereby reducing temozolomide resistance- in glioma cells as part of a new possible therapy.

Fourthly and lastly, Zhang et al. describe, in this Research Topic, an interesting association between TOP2 (nuclear DNA topoisomerase II-alpha) and radioresistance in medulloblastoma. TOP2 acts oncogenically in medulloblastoma, as TOP2A knockdown inhibited cell proliferation, migration, and invasion, whereas overexpression of TOP2A enhanced cell proliferation and invasion. Furthermore, irradiation together with a knockdown inhibitory treatment of TOP2 (si-TOP2) reduced tumorigenicity of medulloblastoma cells, even more than si-TOP2 treatment alone. Therefore, inhibition of TOP2 reduced the radioresistance of medulloblastoma cells.

More research is needed in the field of molecular oncology in order to better understand the role of transcription factors and the epigenetic regulation that contributes to chemoresistance in the most common malignant brain tumors, specifically glioblastoma and medulloblastoma, in adults and children, respectively.

## Author contributions

JC: Writing – original draft, Writing – review & editing. MS: Writing – review & editing. AS: Writing – review & editing.
